# Cholangiocarcinoma Arising from a Type VI Biliary Cyst: A Case Report and Review of the Literature

**DOI:** 10.1155/2015/625715

**Published:** 2015-12-15

**Authors:** İlkay Çamlıdağ, Mehmet Selim Nural, Murat Danacı, İlhan Karabıçak, Kağan Karabulut

**Affiliations:** ^1^Department of Radiology, Ondokuz Mayıs University, Kurupelit, 55139 Samsun, Turkey; ^2^Department of General Surgery, Ondokuz Mayıs University, Kurupelit, 55139 Samsun, Turkey

## Abstract

Cystic dilatations of the cystic duct which are suggested as type VI biliary cysts are very rare and many of them go unrecognized or are confused with other cysts until the operation although they are obvious on imaging studies. They can present with fusiform or saccular dilatations and can be accompanied by common bile duct dilatations. It is important to identify these cysts as they share the same characteristics as the other biliary cyst types and can be complicated with malignancy. We herein present a very unusual case of a cholangiocarcinoma arising from a type VI biliary cyst in a 58-year-old female patient and review the literature. The patient presented with jaundice, weight loss, and abdominal pain. On imaging, the cystic duct and common bile duct were fusiformly dilated and had a wide communication. There was a mass filling the distal parts of both ducts. The patient was urgently operated on after perforation following ERCP. Histopathology was compatible with a type VI biliary cyst and an associated cholangiocarcinoma.

## 1. Introduction

Choledochal cysts are congenital cystic dilatations of any portion of the bile ducts which are not associated with a tumor, stone, or inflammation as the cause of the dilatation [1]. Traditionally, biliary cysts have been classified into five main types by Todani et al. [2]. However, cystic dilatation of the cystic duct is not included in this classification. Congenital cystic duct cysts are extremely rare with a very limited number of cases in the literature. They are regarded as “type VI biliary cysts” and the term was first proposed by Serradel et al. [3]. It is well known that there is an association of bile duct adenocarcinoma and biliary cysts [4]. Adenocarcinoma arising from a type VI biliary cyst is extremely rare with only one reported case in the literature [5]. Herein, we aimed to present the imaging findings of an adenocarcinoma arising from a type VI biliary cyst which is the second case in the literature to our knowledge and review the literature.

## 2. Case Report

**Figure 1:**
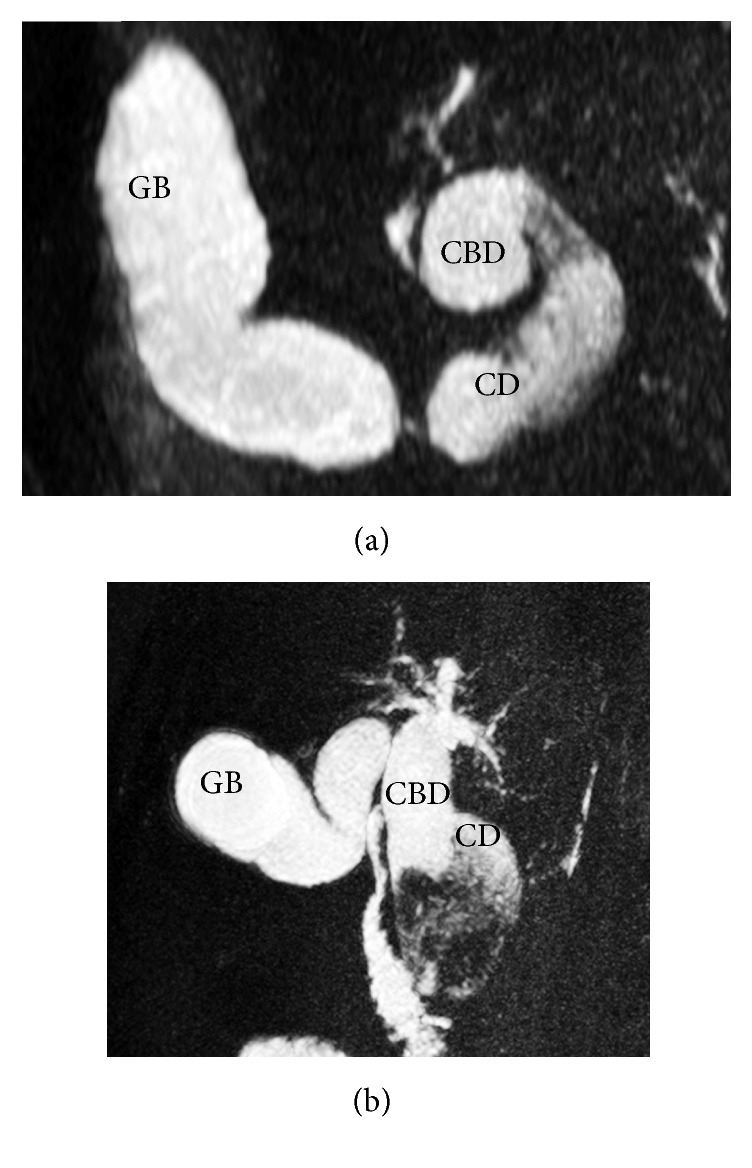
A 58-year-old female with type VI biliary cyst and associated cholangiocarcinoma. (a, b) Axial and coronal MRCP images show fusiformly dilated cystic duct (CD) with a wide opening to the common bile duct (CBD) which is also fusiformly dilated. Intrahepatic bile ducts are slightly dilated in the centre. There is a hypointense, heterogeneous mass filling distal parts of both the cystic and choledochal ducts (GB: gallbladder).

**Figure 2:**
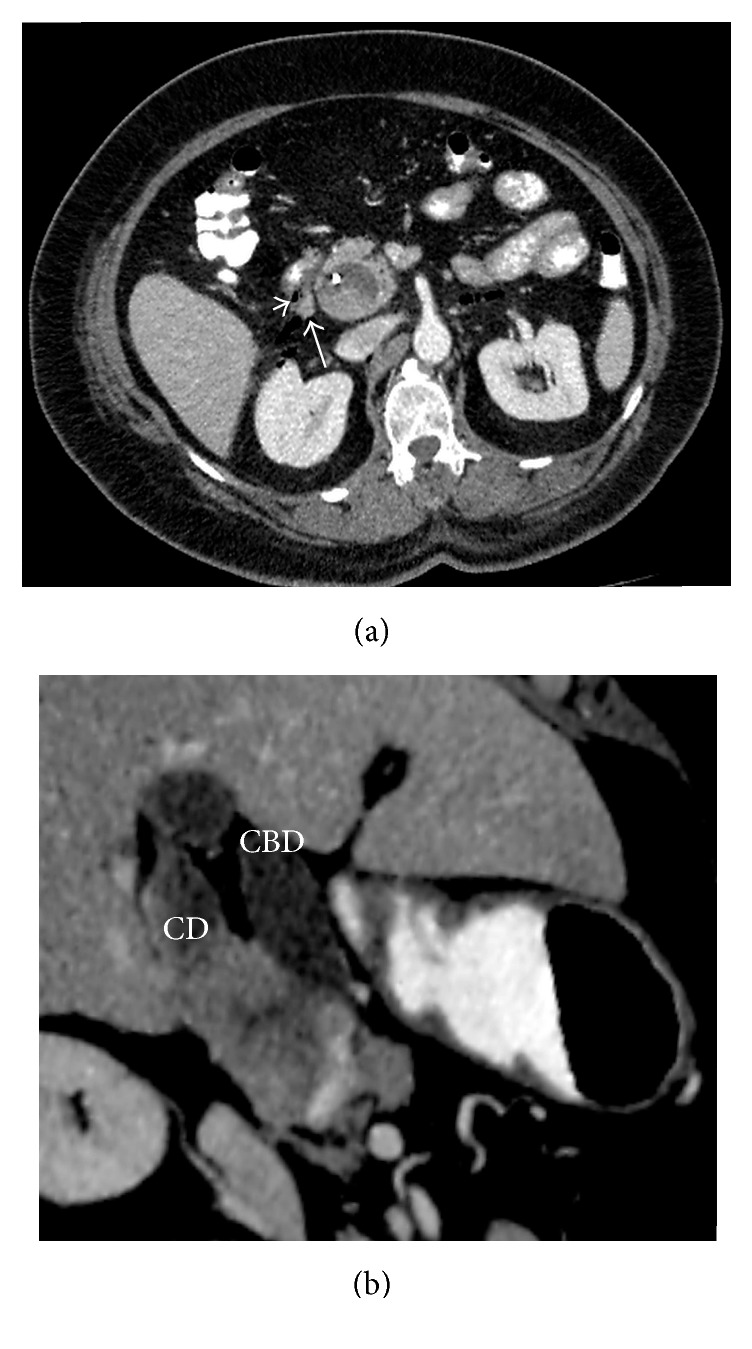
CT examination performed with the suspicion of perforation following ERCP. (a) Axial contrast enhanced CT image shows intraperitoneal free air (short arrow) and periduodenal contrast extravasation (long arrow) suggesting perforation. (b) Multiplanar reconstructed CT image shows fusiform cystic dilatation of the cystic duct having a wide opening to the extrahepatic bile duct which is also fusiformly dilated and the enhancing mass.

**Figure 3:**
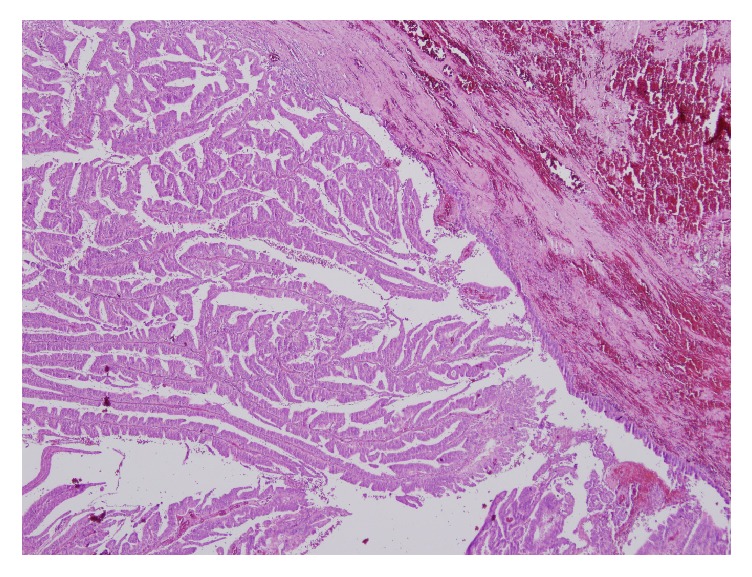
Atypical epithelial cells lining a fibrovascular core extending from the cyst wall that is lined by columnar epithelium are seen on the histopathological specimen.

A 58-year-old female was referred to our hospital from a local hospital. She had complaints of abdominal pain, jaundice for the last two months, and weight loss. On clinical examination, she had slight icteric sclera and tenderness in the right upper quadrant on palpation. Laboratory studies showed markedly elevated hepatic enzymes. Aspartate transaminase, alanine transaminase, and gamma-glutamyl transferase levels were 259, 412, and 1289 IU/L, respectively. Bilirubin levels were slightly elevated with a direct bilirubin level of 1.2 mg/dL and total bilirubin level of 2.61 mg/dL. Other blood tests were unremarkable. On subsequent ultrasound examination, cystic and choledochal ducts were dilated. There was a polypoid mass at the junction of cystic and choledochal duct. The patient underwent magnetic resonance cholangiopancreatography (MRCP) for better delineation of the ultrasonography findings. On MRCP, cystic duct was fusiformly dilated having a wide opening to the common bile duct which was also fusiformly dilated. There was a hypointense, heterogeneous mass filling the distal parts of both ducts. Intrahepatic bile ducts were minimally dilated in the central part but were in normal caliber in the remainder (Figure 1). An endoscopic retrograde cholangiopancreatography (ERCP) procedure was performed for mass sampling. ERCP confirmed the findings of MRCP. After the procedure, the patient complained of severe abdominal pain and underwent computed tomography (CT) which showed free intra-abdominal air and periduodenal contrast leakage (Figure 2(a)) consistent with perforation along with the other findings (Figure 2(b)). There was no sign of lymphadenopathy or distant metastasis on CT. The patient urgently underwent Whipple procedure. It was not until then that all the findings of fusiform dilatation of the cystic and choledochal ducts were interpreted as a cystic duct cyst which was proposed as a type VI biliary cyst. On histopathological examination, there was a polypoid mass which was characterized by atypical epithelial cells lining a fibrovascular core extending from the cyst wall that was lined by columnar epithelium (Figure 3). The histopathological diagnosis of the polypoid mass was consistent with cholangiocarcinoma. To our knowledge, this is the second case of cholangiocarcinoma arising from a type VI biliary cyst.

## 3. Discussion

Biliary cysts are cystic dilatations of the bile ducts and are rarely seen with an incidence of 1 : 100000 to 150000 in western countries and an increased incidence of 1 : 1000 in Asian population. It comprises 1% of all benign biliary diseases [4]. Traditionally, biliary cysts are classified into five categories as described by Todani et al. Type I is cyst of the CBD; type II is a cystic diverticulum of the extrahepatic bile duct; type III is a cyst in the intraduodenal portion of the common bile duct; type IV refers to multiple cysts in the intra- and extrahepatic biliary tract; and type V is single or multiple cysts in the intrahepatic ducts alone which is known as Caroli disease [2]. Although not included in this classification of biliary cysts, cystic dilatation of the cystic duct can also be encountered; however, it is extremely rare with very limited number of reports in the literature mainly consisting of single case reports and only one review to our knowledge [5]. Serradel et al. were the first to propose calling these cysts as type VI biliary cysts [3]. Due to very little acquaintance with the condition, diagnosis is challenging with most of the cases being undiagnosed as our case or misdiagnosed as other types [3, 6, 7]. The correct diagnosis was made intraoperatively in most of the cases in the literature [8].

**Figure 4:**
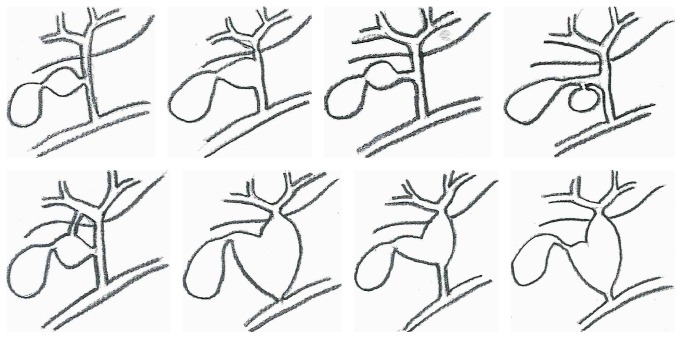
Schematic illustration of the type VI biliary cysts reported in the literature to date.

Type VI biliary cyst can be encountered in different forms. The cystic dilatation can be limited to the cystic duct either fusiform or saccular without any associated findings in the other bile ducts [3, 5, 6, 9, 10] or can be accompanied by fusiform common bile duct dilatation with varying size of communication between the cyst and dilated CBD (see [5, 10, 11], Figure 4).

The most common theory for the development of biliary cysts is the anomalous pancreaticobiliary junction (APBJ). In the APBJ the biliary and pancreatic duct join proximal to the sphincter of Oddi forming a long common channel that causes the pancreatic enzymes to flow upstream into the bile duct resulting in the weakening of the CBD wall and leads to the formation of a cyst. However, not all biliary cysts are accompanied by APBJ and APBJ is also encountered in the absence of biliary cysts. So, more than one mechanism must be responsible for the occurrence of biliary cysts [1].

Presenting features and complications of cysts of the cystic duct are similar to other types of biliary cysts. They might be asymptomatic and incidentally detected or present with varying degrees of abdominal pain, jaundice, and complications such as cholangitis, calculus disease, and malignancy with the last one being the most serious and feared one [1, 4]. Most of the reported cases of biliary cyst associated cancers are cholangiocarcinomas arising from the cyst itself. However, they also can arise from the gallbladder or anywhere else in the biliary ducts especially in the presence of APBJ [1, 12, 13]. To our knowledge, there is only one reported case of cholangiocarcinoma in the literature arising from a type VI biliary cyst reported by Maheshwari et al. [5]. In their case, cholangiocarcinoma arose from a saccular type cystic duct cyst. The information of the presence of APBJ was not given. In our case, the mass originated from the fusiformly dilated cystic and common bile ducts and there was no evidence of APBJ. Two more cases of type VI biliary cyst related cholangiocarcinoma arising from the gallbladder without any APBJ [10] and left hepatic bile duct in the presence of APBJ have also been reported [12].

Radiological imaging is very useful in the diagnosis of biliary cysts. Ultrasonography (USG) is the modality of choice for initial evaluation and frequently provides enough information to make the diagnosis of a biliary cyst. In case of a dilated nonvascular cystic structure near the porta hepatis, attempt should be made to delineate its connection and relationship with the biliary tract and the gallbladder. However, USG may fail to depict these relationships as it is operator-dependent and sensitivity is decreased in the presence of overlying bowel gas and inflammatory conditions. ERCP is the gold standard in the diagnosis of biliary cysts but it is invasive, always requires sedation, and comes with inherent risks such as pancreatitis, perforation, hemorrhage, contrast allergy, cholangitis, and biliary sepsis. Given its relatively moderate risk profile and lower cost, MRCP should be the diagnostic test of choice when preoperatively evaluating biliary cysts and their associated anomalies. MRCP is equivalent to ERCP in determining biliary cyst type and is helpful in diagnosing related pancreaticobiliary anomalies, cholangiocarcinoma, and choledocholithiasis. ERCP should be used when MRCP inadequately visualizes the terminal common bile duct or the pancreaticobiliary junction or when a therapeutic procedure is needed. A multidetector computed tomography (MDCT) with reformatted imaging is another important technique which has the ability to demonstrate the anatomic details of the biliary tree and the ABPJ. It can show the presence of an associated cancer and is useful for staging. The disadvantages are high-dose radiation exposure and contrast agent administration especially in children [1, 4, 5, 9, 13].

Preferred treatment for type VI biliary cysts is the surgical excision as for the other types. Early and proper diagnosis is important to avoid complications and choose the appropriate surgical method. Cholecystectomy and transection of the cystic duct are sufficient in the presence of a narrow common duct junction. However, in the presence of a wide opening to the common bile duct, hepaticoduodenostomy or hepaticojejunostomy should be performed [7, 11].

In conclusion, we had a very typical but unrecognized case of type VI biliary cyst with obvious findings on all imaging modalities. Because of the unfamiliarity with the condition, the cysts were interpreted as dilatations secondary to mass obstruction. However, disproportionate and only mild dilatation of the central intrahepatic bile ducts should have warned us that it was not result of obstruction as they would be much more dilated given the degree of the extrahepatic bile duct dilatation. We think that increased familiarity with cystic duct cysts is crucial as early treatment can prevent complications like inflammation and more importantly malignancy. We think that cystic duct cysts should officially be included in the Todani et al. classification as type VI biliary cyst with all its subtypes because it has all the characteristics of the other biliary cysts in terms of clinical findings and complications.
